# Breast cancer pathology services in sub-Saharan Africa: a survey within population-based cancer registries

**DOI:** 10.1186/s12913-020-05752-y

**Published:** 2020-10-02

**Authors:** Hannes-Viktor Ziegenhorn, Kirstin Grosse Frie, Ima-Obong Ekanem, Godwin Ebughe, Bakarou Kamate, Cheick Traore, Charles Dzamalala, Olufemi Ogunbiyi, Festus Igbinoba, Biying Liu, Marcus Bauer, Christoph Thomssen, Donald Maxwell Parkin, Claudia Wickenhauser, Eva Johanna Kantelhardt

**Affiliations:** 1 Institute of Medical Epidemiology, Biometrics and Informatics, Martin-Luther-University Halle-Wittenberg, Magdeburgerstrasse 8, 06097 Halle, Germany; 2 Department of Pathology, University of Calabar, Cancer Registry, Calabar, Nigeria; 3 University of Calabar Teaching Hospital, Calabar, Nigeria; 4 Department of Pathology, University of Bamako, Bamako Cancer Registry, Bamako, Mali; 5 University of Malawi College of Medicine, Cancer Registry, Blantyre, Malawi; 6 University of Ibadan, Cancer registry, Ibadan, Nigeria; 7 University of Ibadan College of Medicine, Ibadan, Nigeria; 8 National Hospital Abuja, Abuja, Nigeria; 9 The African Cancer Registry Network, INCTR African Registry Program, Oxford, UK; 10 Nuffield Department of Population Health, University of Oxford, Oxford, UK; 11 Institute of Pathology, Martin-Luther-University Halle-Wittenberg, Halle, Germany; 12 Department of Gynaecology, Martin-Luther-University Halle-Wittenberg, Halle, Germany; 13 International Agency for Research on Cancer, Lyon, France

**Keywords:** Pathology, Sub-Saharan Africa, Guidelines, Immunohistochemistry, Population-based cancer registry

## Abstract

**Background:**

Pathologists face major challenges in breast cancer diagnostics in sub-Saharan Africa (SSA). The major problems identified as impairing the quality of pathology reports are shortcomings of equipment, organization and insufficiently qualified personnel. In addition, in the context of breast cancer, immunohistochemistry (IHC) needs to be available for the evaluation of biomarkers. In the study presented, we aim to describe the current state of breast cancer pathology in order to highlight the unmet needs.

**Methods:**

We obtained information on breast cancer pathology services within population-based cancer registries in SSA. A survey of 20 participating pathology centres was carried out. These centres represent large, rather well-equipped pathologies. The data obtained were related to the known population and breast cancer incidence of the registry areas.

**Results:**

The responding pathologists served populations of between 30,000 and 1.8 million and the centres surveyed dealt with 10–386 breast cancer cases per year. Time to fixation and formalin fixation time varied from overnight to more than 72 h. Only five centres processed core needle biopsies as a daily routine. Technical problems were common, with 14 centres reporting temporary power outages and 18 centres claiming to own faulty equipment with no access to technical support. Only half of the centres carried out IHC in their own laboratory. For three centres, IHC was only accessible outside of the country and one centre could not obtain any IHC results. A tumour board was established in 13 centres.

**Conclusions:**

We conclude that breast cancer pathology services ensuring state-of-the-art therapy are only available in a small fraction of centres in SSA. To overcome these limitations, many of the centres require larger numbers of experienced pathologists and technical staff. Furthermore, equipment maintenance, standardization of processing guidelines and establishment of an IHC service are needed to comply with international standards of breast cancer pathology.

## Background

Cancer is a growing burden in sub-Saharan Africa (SSA) due to an increasing life expectancy and growing prevalence of risk factors. Breast cancer is the most commonly diagnosed cancer entity and the second most frequent cause of cancer mortality in the region [1]. Compared to high-income countries, overall survival is much shorter due to higher proportions of late-stage disease [2–4] and limited diagnostic and therapy options. While several studies have highlighted the need for early detection of breast cancer and downstaging to improve survival [5, 6], only a few studies have investigated the current capabilities of diagnostic and treatment services to provide a continuum of care. A recent study from Burkina Faso estimated a current annual unmet need of diagnostic services for 184,562 women with breast-related symptoms and pointed to the insufficient availability and quality of diagnostic services in SSA [7].

Several studies mention the need for an improved pathology service. A recent Lancet series highlighted the state of pathology and laboratory medicine in low-income countries [8–10]. Accessible, reliable, high quality and cost-effective pathology takes centre stage in triaging women with breast-related symptoms in order to provide optimal and timely treatment. Depending on the pathology tier, laboratories should provide adequate equipment, skilled manpower and infrastructure for communication, documentation and storage to ensure that the capabilities for fine-needle aspiration cytology (FNAC), tissue biopsies/surgical excisions and tissue preparation, processing, haematoxylin-and-eosin staining and interpretation are guaranteed for each patient. The provision of an affordable immunohistochemistry (IHC) technique, eventually with the assistance of partner laboratories, should be part of the package [11]. Pathology procedures in low- and middle-income countries should follow guidelines and quality assurance procedures [12]. Therefore we performed this survey to assess the current status of essential components of the pre-analytic-, analytic-phase (e.g. timely specimen handling, cool chain, availability of buffered formalin, paraffin wax, freezer storage for antibodies and continuous supply of consumables and reagents) and post-analytic-phase (e.g. pathology reporting, storage of reports and formalin-fixed paraffin embedded-blocks). Benchmarks relevant to breast cancer pathology, which would be desirable for centres included in this study are given in Table 2.

Previous surveys have already highlighted huge challenges and unmet needs for the pathology services in SSA [13]. The majority of laboratories in SSA fall far short from accreditation standards like the ISO 15189, the Clinical-Laboratory standards of the International Society for Standardization [14]. Therefore the WHO-Africa regional office (WHO-AFRO) established the Stepwise Laboratory Quality Improvement Process Towards Accreditation (SLIPTA) [15]. In a study from Kampala (Uganda) only 5 % of the laboratories even reached the first step in this five-step process to international accreditation [16]. In 2014 out of 49 countries in SSA, 37 had no laboratory accredited to international quality standards [17]. Absence or low density of pathologists, inadequate equipment and power supply in laboratories and unaffordability of services for patients were reported for most countries of SSA. According to a 2013 study, there were 755 pathologists working in SSA, more than half of them only in Nigeria and South Africa [18]. The pre-analytical phase is also fundamental for a quality breast cancer diagnostic; shortcomings can easily lead to tissue degeneration and poor quality formalin-fixed paraffin-embedded (FFPE) blocks and slides. In SSA, long ischaemic times (time between surgical extraction and formalin fixation), uncontrolled high-temperature exposure, inadequate long or short fixation times and the use of unbuffered formalin could have a major influence on the IHC quality. Reliable IHC results are essential to make therapy decisions based on hormone receptor status [19]. For patients with estrogen receptor positive tumors, Tamoxifen has a high impact on survival [20].

While a rough nationwide survey of general pathology services gives an initial overview of the current situation in SSA [18], detailed information from individual pathology centres on the capabilities, procedures and barriers to providing quality services for breast cancer diagnosis is needed to further describe the current situation of pathological breast cancer management in SSA. The aim of this study was to describe the staff, workflow, equipment and services available at selected pathology centres within the geographical area of population-based cancer registries (PBCRs) in SSA. The results were interpreted in relation to the defined population and incidence of breast cancer in the cancer registry area. We aimed to identify detailed problems and deficiencies, especially in the establishment and provision of IHC services, as an essential prerequisite for improvements towards high quality breast cancer care, including personalized endocrine treatment.

## Methods

A short description of the study and a questionnaire was sent to all members (24) of the African Cancer Registry Network (AFCRN) in December 2016 by email. The replies were collected both in person and via email. Breast cancer incidences for 2015 were available from GLOBOCAN [1]. Data from the underlying population were provided by the respondents from their respective National statistical authorities for 2015. Those are projections from the most recent census. If available, data was collected for 2015. If not available, the most recent population data before 2015 was used and for the breast cancer incidence an average case number was calculated from years before. The questionnaire was developed in collaboration with the African Cancer Registry Network and pretested among other German and African pathologists. Minor changes were made to improve understanding and the questionnaire was provided in both English and French languages.

The questionnaire enquired about secondary data from health facilities: procedures within the responding pathology centre and also general information about services within the population-based cancer registry area. The questions about procedures were framed as usual practice. The questions covered the following sections: procedures of the pre-analytical phase (e.g. time to fixation, transportation conditions, fixation time, formalin quality, paraffin quality); diagnostic and technical capabilities (refrigerator, 4–8 °C; freezer, below 0 °C); availability of IHC; documentation and storage. The data was processed and descriptive statistics were calculated with IBM SPSS Statistics for Windows, Version 25.0.

To illustrate the procedures between specimen removal and pathological analysis, only the longest transportation time and the most unfavourable transportation condition were included in cases of multiple answers. The multiple centers in Nigeria and Uganda each operated independently.

The African Cancer Registry Network is a project of the Cancer Registry Programme of the International Network for Cancer Treatment and Research (INCTR). It was inaugurated in 2012 and has currently 35 members out of 25 countries from SSA. As partner of the International Agency for Research on Cancer (IARC) within the framework of its Global Initiative for Cancer Registry Development in Low- and Middle-Income Countries, it provides technical and scientific support to countries, delivers training in population-based cancer registration and coordinates international research projects. The network provides data for GLOBOCAN [1, 21].

## Results

From 24 invited cancer registries, 20 registries from 17 different countries participated in the survey. There were no major differences between the responding and non-responding registries concerning region, per-capita income and size of the registries. There are registries from low income countries as well as from high income countries (Seychelles, Mauritius) included in this study. All respondents were pathologists, apart from two clinicians and one cancer registrar, they were all head of the department or the registry. In Table 1, general information about the participating pathology centres and the related population-based cancer registry areas is described. Annual breast cancer incidence within the cancer registries ranged between 35 and 500, processed by 1–15 pathologists in the different registry areas. The population per pathologist ranged from 30,000 (Seychelles) to 1,830,000 (Republic of the Congo). On average we found a population of 392,000 per pathologist. The pathology centres handled between 10 (Mozambique) and 386 (Mauritius) cases. Lack of IHC for the breast cancer specimen was a common problem. The annual unmet need of IHC tests totalled 1416 for pathology centres with no IHC in their own laboratory and 887 for centres with no IHC laboratory in their whole area. A number of 3365 breast cancer patients had access to IHC within their registry area. We found that centres with IHC available served between 35 and 500 BC cases per year. We assumed that availability of one or more centres performing IHC is sufficient for all cases from the registry (amounting to 0,7–9,4 per week).

**Table 1:** General information about pathology centres

Country	Cancer registry location	Population^1^	BC cases	Pathology Facilities	Pathologists	Population per pathologist	Laboratories with IHC	Oncologist	Radiotherapy	BC Cases^3^	Breast tumor board	IHC
		In the cancer registry area								In the pathology centre		
**Benin**	Cotonou	679.000^e^	75^2^	5	4	170.000	1	Yes	No	53	Yes	Yes
**Ethiopia**	Addis Ababa	3.050.000^d^	450	8	27	113.000	1	Yes	Yes	225	Yes	Yes
**Ghana**	Kumasi	2.173.000^c^	59^2^	6	5	435.000	3	Yes	Yes	47	Yes	Yes
**Guinea**	Conakry	1.668.000^f^	45^2^	2	3	556.000	0	Yes	No	16	Yes	No
**Ivory Coast**	Abidjan	4.707.000^f^	500	5	12	392.000	2	Yes	No	300	Yes	No
**Kenya**	Eldoret	894.000^b^	88	3	5	179.000	3	Yes	No	75	No	Yes
**Malawi**	Blantyre	952.000^g^	43	1	3	317.000	0	Yes	No	37	No	No
**Mali**	Bamako	1.810.000^b^	200	1	5	362.000	0	Yes	Yes	160	Yes	No
**Mauritius**	Mauritius	1.263.000^g^	483	4	11	115.000	2	Yes	Yes	386	Yes	Yes
**Mozambique**	Beira	458.000^e^	16^2^	1	3	153.000	0	No	No	10	No	No
**Nigeria**	Ibadan	2.549.000^a^	366	3	7	364.000	1	Yes	Yes	293	Yes	No
**Nigeria**	Abuja	2.442.000^g^	220	5	9	271.000	6	Yes	Yes	110	Yes	Yes
**Nigeria**	Calabar	2.893.000^g^	154	3	6	482.000	1	Yes	No	154	Yes	No
**Republic Congo**	Brazzaville	1.830.000^f^	234	1	1	1.830.000	1	Yes	No	234	Yes	Yes
**Seychelles**	Seychelles	90.000^e^	35	1	3	30.000	1	Yes	No	33	No	Yes
**Sierra Leone**	Free Town	1.056.000^g^	40	1	1	1.056.000	0	No	No	16	No	No
**Swaziland**	Swaziland	1.104.000^g^	93	1	2	552.000	0	Yes	No	93	Yes	No
**Uganda**	Gulu	762.000^f^	124^2^	1	4	191.000	1	Yes	No	112	No	No
**Uganda**	Kampala	2.376.000^c^	84	8	15	158.000	3	Yes	Yes	42	Yes	Yes
**Zimbabwe**	Harare	1.469.000^d^	493	5	9	163.000	2	Yes	Yes	296	Yes	Yes

*Abbreviations*: *BC* Breast Cancer, *IHC* Immunohistochemistry

^1^Most recent population (^a^2006, ^b^2009, ^c^2010, ^d^2012, ^e^2013, ^f^2014, ^g^2015), nearest 1000

^2^Case number for 2015 was not available, as a substitute, the average number of cases in other years was calculated:

Benin (2013–2015), Ghana (2012), Guinea (2001–2010), Mozambique (2019–2013), Uganda (2008–2012)

^3^Number was calculated from the estimated proportion of the total number in the cancer registry area

**Fig. 1:**
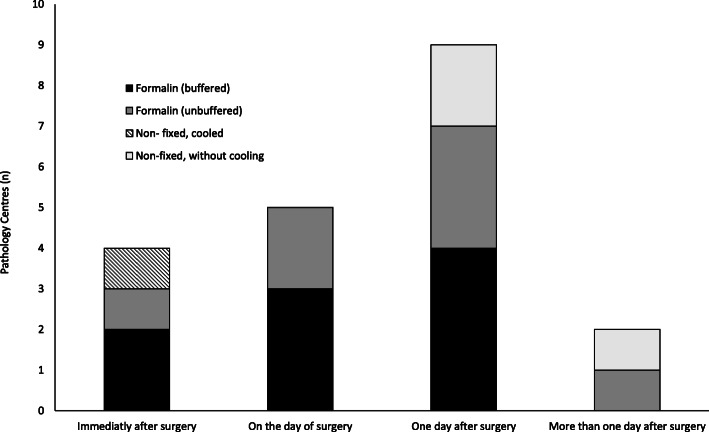
Timing and conditions of specimen transport to the pathology centre (*n* = 20 centres). The multiple centres in Nigeria and Uganda each operated independently

Time between specimen removal and arrival at the pathology facility and the condition of transport are described in Fig. 1. Only one respondent described mastectomy specimens being sliced by a surgeon before formalin fixation. Buffered formalin was used in 50% (10) of the centres and unbuffered in the rest. All centres used 10% concentrated formalin. Dehydration and paraffin penetration were performed in 12 centres automatically but only partially or manually in the others. Tissues were incubated overnight in six (30%) centres, for about 48 h in five (25%) centres and for more than 48 h in eight (40%) centres; the standard incubation time is 48 h. When adding transportation time for the latter, six (30%) of the centres had tissue in formalin for longer than 72 h. Formalin-fixed paraffin-embedded blocks were prepared with pure paraffin in 14 (74%) centres, with paraffin and additives in three (16%) and with candle wax in two (11%).

All centres processed fine-needle aspirations and surgical biopsies. In 16 (80%) centres, core needle biopsies were performed. Fine-needle aspiration was the most common diagnostic practice, with 10 (50%) of all centres performing it at least once a day; core needle biopsy was only performed in five centres on a daily basis.

**Fig. 2:**
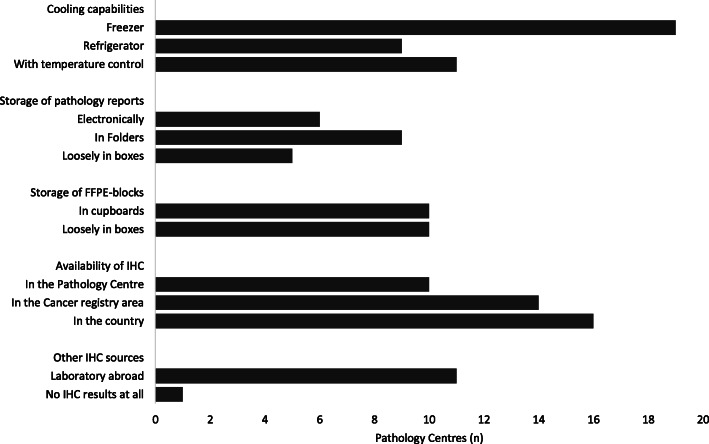
Cooling, storage and immunohistochemistry capabilities. FFPE, formalin-fixed paraffin-embedded; IHC, immunohistochemistry. The multiple centres in Nigeria and Uganda each operated independently

The different cooling (for IHC antibodies), storing, documentation and IHC capabilities are presented in Fig. 2. Eighteen centres (90%) store their formalin-fixed paraffin-embedded blocks for 5 or more years and one centre for only 1 year. Only three centres (15%) had air conditioner in the block storage room. The following difficulties were reported: in 14 (70%) centres there were periods without electricity; in 18 (90%) centres there was faulty equipment, such as processing machines, microtomes or microscopes, with no technical support available; and in all centres with IHC there were delays in the supply of antibodies and reagents during the last 2 years. In 14 (70%) of the centres with IHC the delay in supply of consumables was more than 1 month, extending to more than 1 year in some.

In the 10 centres where IHC was provided within their own laboratory, oestrogen and progesterone receptor analysis was available. Eight of these centres also provided HER2 (human epidermal growth factor receptor 2) analysis and five provided Ki67 analysis. For three (15%) centres IHC was only performed outside the country and in one (5%) it was not possible to obtain any IHC results. However, in 10 (50%) of the centres without IHC, technicians trained in IHC procedures were available. The out-of-pocket payment to obtain IHC analysis varied between 0 and 200 US dollars. Eleven centres reported that they sent specimens abroad for IHC, to India, France, South Africa and other countries. The price per case for transporting samples to another laboratory abroad varied between 10 and 300 US dollars.

For 16 (80%) of the 20 centres there was an oncologist available in the same hospital, in another two centres the oncologist was located in the same or another city and for the remaining two centres there was no oncologist available. Radiotherapy was accessible for patients in eight (40%) of the centres but for twelve (60%) it was not. A breast cancer tumour board regularly took place in 13 (65%) of the centres.

## Discussion

A stable, comprehensive, guideline-oriented and financially accessible pathology service should be the basis for high quality diagnosis and treatment of breast cancer in SSA. There are National Cancer Control Network (NCCN) guidelines available, harmonized for SSA, and standardized pathology protocols from the College of American Pathologists [19]. A four-tier-based system has been recommended for low-income countries [11]: laboratories covering regional care or national pathological care are considered as tier 3 or tier 4, respectively. Pathology services at the different tiers should complement each other in their areas of responsibility, whereas tier 3 and tier 4 laboratories are equipped with more specialized staff, have better equipment and provide more complex tests. Based on this tier model, the pathology laboratories included in this study should provide at least tier 3 or tier 4 compliant services, based on their size, location and patient case numbers. Therefore they represent a group centres in large cities, probably best-equipped in their countries where the rest of SSA is probably even less equipped. However, the results of our study indicate that the majority of these laboratories were unable to provide those minimum services required due to shortcomings in staff, equipment and diagnostic options and would rather be classified as tier 2 (Please see Table 2).

**Table 2:** Requirements for breast cancer pathologies depending on the tier according to Sayed S. et al. [9], and Fleming KA et al. [11]

	Tier 2^1^	Tier 3/ Tier 4 (Includes Tier 2 Capabilities)^2,3^
**Population served**	50,000–200,000	3–6 million
**Staff**	1 general pathologist, 6 laboratory technicians and pathology assistants	4 pathologists, 2 clinical scientists, 20 technicians and pathology assistants
**Communication infrastructure**	Paper, electronic, or (preferred) laboratory information system	Electronic or laboratory information system
**Diagnostic**	Anatomic pathology: FNAC, tissue biopsies, and surgical excisions-processing, H&E stain, and interpretation	Anatomic pathology: special stains, including immunohistochemistry
**Public health**	Report to other registries	Hospital-based registry/ population-based registry
**Accreditation**	Progressing towards accreditation, such as SLIPTA	National or international accreditation, such as ISO 15189 or other
**Equipment**	FNAC and biopsy fixation, microscope, tissue processor, microtome for anatomic pathology, refrigerator	Automated tissue processor, immunohistochemistry station, freezer

^1^ Tier 2: Laboratories in district hospitals that receive specimens from their own patients and receive referrals from tier 1 facilities

^2^ Tier 3: Laboratories in regional or provincial hospitals that receive specimens from their own patients and receive referrals from tier 1 and 2 facilities

^3^ Tier 4: Laboratories in national or teaching hospitals that receive specimens from their own patients and receive referrals from tier 1, 2 and 3 facilities

*Abbreviations*: *FNAC* Fine-needle aspiration cytology, *H&E* Hematoxylin and Eosin, *ER* Estrogen, *PR* Progesterone, *SLIPTA* Stepwise Laboratory Quality Improvement Process Towards Accreditation

Optimized, standardized handling of breast cancer tissue before processing is essential to acquire reliable results for later diagnostics, especially IHC. We found significantly prolonged times and exposure to high temperatures during transport between the operation room and the pathology laboratory, which may lead to tissue and protein degradation for large, non-sliced samples. This should be avoided, especially when use of IHC is planned. To ensure reliable IHC results, standard procedures should be established in each centre to meet both minimum and maximum tissue fixation times because periods of several days, depending on the IHC procedure, may inevitably limit the reliability of the results [22]. The use of un-buffered formalin is also problematic. In the case of larger mastectomies, slicing should be carried out by the surgeon in order to ensure sufficient fixation at a penetration rate of 1 mm/h of formalin, but 95% of respondents did not practice this in their centre. Alternative substances, such as candle wax, or additions to the paraffin limit the quality of the formalin-fixed paraffin-embedded blocks and make further processing difficult.

Fine-needle aspiration cytology is the most commonly used invasive diagnostic procedure among the centres surveyed and was performed on a daily basis. The process is cost effective and requires minimal equipment. Although 80% of centres reported the use of core needle biopsy, only 25% mentioned performance on a daily basis. In another study, 85% of surveyed centres in SSA reported breast cancer diagnosis by core needle biopsy [23]. The optimal invasive diagnostic method might differ between the different settings. The sensitivity of fine-needle aspiration cytology is limited and thus core needle biopsy would improve diagnosis. The problem is that core needles are still rather costly in this setting. Even the use of core needle biopsy in the pathology department might be problematic due to the lack of ultrasound guidance and therefore, integration into surgical or clinical management could be attempted. However, to establish IHC, routine core needle biopsy is desirable compared with fine needle aspiration [24].

As a further prerequisite for the establishment of IHC, cold storage of antibodies required for the test must be available. This requires constant temperature control with the appropriate technology. Regular power outages for the individual centres are a big challenge for SSA, requiring a separate power source even though this is associated with high costs. A well-known problem is the lack of technical support for centres in the region. This is one of the critical issues when it comes to maintenance of pathology services in the long term. The lack of repair options leads to unexpected failures and enormous costs for the individual centres. A greater willingness of the companies responsible for providing the appropriate support is desirable.

Well-structured reporting and archiving is important for both clinical work and later studies. Unfortunately, good standards in this field are not common. This may lead to less reliable results and possibly the wrong scientific conclusions. In clinical retrospective studies in the region, in some cases only around 50% of the patient records or formalin-fixed paraffin-embedded blocks were found and classified [25, 26] due to storage of reports and blocks loose in boxes. In our study 5 centres stored their pathology reports and half of the centres their formalin-fixed paraffin embedded-blocks loosely in boxes. Six of the responding centres documented their pathology reports electronically. Although electronic data collection is desirable, it is not necessarily the ideal solution. With simple options such as structured cabinets or folders much could be achieved.

A broad review and meta-analysis of hormone receptor status in SSA (April 2014) found only 26 studies reporting oestrogen receptor status in patients from SSA [27]. Since then, other publications have reported scientific collaborations with IHC done abroad or temporary successful technology transfer of IHC testing in pathology centres of SSA. Most studies aim to reveal insight into tumour biology in Africa. There are reports from Ghana [28, 29], the Republic of Congo [30], Kenya [31], Tanzania [32, 33] and Ethiopia [34]. Some efforts were also made to publish results from established IHC services locally, with experiences reported from the Ivory Coast [35], Nigeria [36], Uganda [25], Angola [37], Ethiopia [38] and others. Notably, we did not find any publications reporting on the actual challenges, difficulties and pitfalls when trying to establish IHC in pathology centres of SSA.

Vanderpuye and colleagues [23] surveyed 19 centres in 14 countries breast cancer management. The results showed that 42% of all centres offered IHC in their laboratory, 37% within the country and 21% outside the country. All centres reported a bottleneck in obtaining the required reagents and antibodies. It is notable in our study that 55% of all centres received IHC results from abroad; this indicates that in patients financially able to afford it, the diagnostic services take place abroad. This management is not sustainable, may lead to dependencies and hinders the development of pathological services. It also leads to high costs that are affordable for only a small proportion of patients, as the cost of IHC was reported to be more than twice the monthly gross national income per capita in some countries [39].

This study provides a detailed picture of pathology services in SSA compared with earlier studies, due to its linkage with population-based cancer registries. For example, in our study we found a total of 19 pathologists working within the two urban cancer registries in Uganda (Kampala and Gulu). The population in these areas is about 2.8 million people, resulting in one pathologist per 148,000 population. This is a low coverage compared to European countries where inhabitants per one pathologist varies between 14,309 (Island) and 63,028 (Poland) and is on average 32,018 [40]. According to data from a 2016 study, there are 24 pathologists working in the whole of Uganda. This means with 19 pathologists are working in the population-based cancer registry areas included in our study, only five pathologists remain for the rest of the country with 32 million inhabitants, resulting in only one pathologist per 6.4 million in the rural population [18]. On average, in our urban population-based cancer registry areas, we found a population of 392,000 per pathologist. Previous studies for the whole of Africa found an average population of more than 1 million per pathologist [13]. The relatively good average ratio can be explained by the choice of the larger surveyed centres. This reveals the huge disparity in pathology service within Africa, with considerable coverage in urban areas but a very high unmet need in rural areas.

Breast cancer diagnostic caseloads of between 16 and 386 per year suggest further centralization of the service in some locations to maintain a high quality (the recommended caseload is 150 per year). An annual need for IHC between 35 and 500 cases seems achievable by single-slide staining of those with lower caseloads and using automated staining machines for higher caseloads. Notably, the logistics to optimize the use of antibodies, usually sold in batches for 50 or more, becomes difficult when caseloads are low.

The results of this study are still likely to overestimate the pathology services in SSA for the majority of patients as the pathology centres within the population-based cancer registries are virtually all in urban areas (except for the national cancer registries in Mauritius, the Seychelles and Swaziland) and within large cities (except Beira in Mozambique, which has a population of less than 500,000). Thus, we expect a far lower coverage in most rural areas of the countries. The service to the rural population through urban centres is difficult to assess on a true population-based level. Although large urban centres should be accessible nationwide for patients, this is often too difficult for many patients due to a lack of infrastructure and high travel costs [41]. However, the number of patients receiving care from these large urban centres is higher than estimated from the catchment areas of the registries: a study from the only oncology centre in Ethiopia at Addis Ababa found that around half the patients served were from out of the city [42].

Another limitation of this study is the small number of centres surveyed. Only pathology centres with an affiliated population-based cancer registry were contacted, which led to the inclusion of rather large and only governmental facilities. The situation of equally important private as well as primary care served by smaller laboratories could not be determined due to the study design. However, the shortfall in public care providers already points towards drastic restriction of the service for cancer patients. The time between taking the specimen and reporting the result plays a key role in medical decision-making and would have required a prospective survey, therefore this was not assessed here.

## Conclusions

This study shows that pathology services in SSA are facing unique challenges that need to be addressed urgently in order to improve breast cancer diagnosis and care in the region. The current number of trained staff cannot possibly meet the need of a growing population demanding service. Since breast cancer is the most commonly diagnosed cancer and the second most frequent cause of cancer mortality in SSA, the National Cancer Control Network released guidelines to standardize breast cancer care and early detection of malignancy. According to the guidelines pathology service with reliable diagnostic service is essential. Adequate numbers of trained staff could be accomplished by upscaling local postgraduate training of pathologists and technicians. To provide the highly effective systemic endocrine treatment with tamoxifen as a low-cost, well-tolerated and cheap medication for adjuvant and also palliative purposes, hormone receptor testing is required [19]. Reliably establishing IHC or an alternative method to determine receptor status is obviously a challenge. Pre-analytical and analytical procedures (e.g. timely specimen handling, cool chain, availability of buffered formalin, paraffin wax, freezer storage for antibodies and continuous supply of consumables and reagents) need to be in place and affordable. Furthermore, consumables and antibodies should be made available at low cost for centres with lower caseloads that do not offer the service. Finally, population-based cancer registries that include patient, pathology, therapy and outcome data can provide evidence of improving care and survival over time. Further efforts should be made to gain insight into the current state of rural as well as private pathology services in the region not covered by this study. Important aspects would be the affordability and accessibility of basic pathological care at population level. It would also be helpful to provide reports of successful implementation of IHC in low-income countries to encourage wider-spread utilization.

## Data Availability

The datasets used and/or analysed during the current study are available from the corresponding author on reasonable request.

## Abbreviations

IHC: Immunohistochemistry
SSA: Sub-Saharan Africa
